# Orbital-effect-induced finite-momentum pairing and Josephson vortex lattice melting in layered Ising superconductors

**DOI:** 10.1093/nsr/nwag084

**Published:** 2026-02-10

**Authors:** Hongyi Yan, Haiwen Liu, Yi Liu, Ding Zhang, Xincheng Xie

**Affiliations:** Center for Advanced Quantum studies, School of Physics and Astronomy, Beijing Normal University, Beijing 100875, China; Key Laboratory of Multiscale Spin Physics, Beijing Normal University, Beijing 100875, China; Center for Advanced Quantum studies, School of Physics and Astronomy, Beijing Normal University, Beijing 100875, China; Key Laboratory of Multiscale Spin Physics, Beijing Normal University, Beijing 100875, China; Interdisciplinary Center for Theoretical Physics and Information Sciences, Fudan University, Shanghai 200433, China; Department of Physics and Beijing Key Laboratory of Opto-electronic Functional Materials & Micro-nano Devices, Renmin University of China, Beijing 100872, China; Key Laboratory of Quantum State Construction and Manipulation, Renmin University of China, Beijing 100872, China; State Key Laboratory of Low Dimensional Quantum Physics and Department of Physics, Tsinghua University, Beijing 100084, China; Beijing Academy of Quantum Information Sciences, Beijing 100193, China; Frontier Science Center for Quantum Information, Beijing 100084, China; Interdisciplinary Center for Theoretical Physics and Information Sciences, Fudan University, Shanghai 200433, China; International Center for Quantum Materials, School of Physics, Peking University, Beijing 100871, China; Hefei National Laboratory, Hefei 230088, China

**Keywords:** layered Ising superconductor, orbital-FFLO state, Josephson vortex, Josephson vortex lattice melting

## Abstract

The Fulde–Ferrell–Larkin–Ovchinnikov (FFLO) state is a fascinating superconducting phase characterized by a spatially modulated order parameter that occurs in unconventional superconductors under strong magnetic fields. In this study, we explore the impact of in-plane magnetic fields on the superconducting state in layered Ising superconductors. Our findings reveal the emergence of an orbital-effect-induced finite-momentum pairing state that is coupled to Josephson vortices and is fundamentally different from the FFLO state. Notably, recent experiments have revealed an unexpected first-order phase transition in bulk Ising superconductors. Our theoretical analysis demonstrates that this phase transition is primarily driven by the melting of a Josephson vortex lattice. Furthermore, our calculations for both the in-plane upper critical field and the melting line are in excellent agreement with the experimental data. These results not only advance the understanding of novel field-induced phases in layered Ising superconductors, but also pave the way for controlling these exotic states through magnetic-field manipulation.

## Teaser

This study reveals that bulk layered Ising superconductors can host finite-momentum pairing states (orbital FFLO states) under in-plane magnetic fields, and establishes a quantitative method to compute the Josephson vortex lattice melting line, providing a theoretical basis for understanding high-field vortex phase transitions.

## INTRODUCTION

The interplay between the superconducting phase and magnetic flux has led to significant quantum effects, such as flux quantization [1] and the Fraunhofer diffraction pattern of supercurrent in Josephson junctions [2]. Layered superconductors represent a new frontier for exploring the interplay between the superconductivity and magnetic flux. In these materials, the Josephson vortex lattice arises from the interplay between interlayer Josephson tunneling and the magnetic vector potential, resulting in a periodic arrangement of vortices in response to an applied magnetic field [3–9]. While the melting of the Abrikosov vortex lattice has been extensively studied both theoretically [10–20] and experimentally [21–29], the behavior of the Josephson vortex lattice in layered superconductors under in-plane magnetic fields remains insufficiently explored [30–34], with a notable lack of systematic studies and quantitative criteria.

In conventional superconductors, magnetic fields can affect superconductivity in two main ways. First, they cause energy splitting between spin-up and spin-down electrons (the Zeeman effect), leading to Cooper-pair destruction when the Zeeman energy approaches the superconducting energy gap. This sets the so-called Pauli paramagnetic limit for the upper critical field, denoted as $B_{\rm P} = 1.86 \, T_{\rm c}$, where $T_{\rm c}$ is the superconducting transition temperature [35,36]. Second, magnetic fields can also break Cooper pairs by disturbing the motion of electrons through what is known as the orbital effect, making it harder for electrons to stay paired; its strength is characterized by the orbital-effect limiting field $B_{\rm orb}$. The competition between these two effects is quantified by the Maki parameter, $\alpha _{M} = \sqrt{2} B_{\rm orb}/B_{\rm P}$. In most conventional superconductors, the orbital effect dominates over the Zeeman effect, resulting in $\alpha _{M}<1$. In the 1960s, Fulde, Ferrell, Larkin and Ovchinnikov proposed a finite-momentum configuration of Cooper pairs, known as the Fulde–Ferrell–Larkin–Ovchinnikov (FFLO) state [37,38]. This state is driven by the Zeeman effect and can exist under magnetic fields stronger than the Pauli limit. To observe the FFLO state, the orbital effect must be sufficiently weak, which requires a large Maki parameter. To date, evidence of the FFLO state has been reported only in heavy-fermion systems [39–42], organic superconductors [43–46], FeSe [47,48] and tricolor superlattices [49].

In transition-metal dichalcogenide (TMD) materials, the absence of local spatial inversion symmetry gives rise to strong Ising spin-orbit coupling (SOC), which locks the paired spins in the out-of-plane direction. This effect suppresses the Zeeman response to the in-plane magnetic field, resulting in high in-plane upper critical fields in these materials [50–53]. In this case, the magnetic field primarily influences the superconducting states through the magnetic vector potential, also known as the orbital effect [54]. While this effect is negligible in single-layer Ising superconductors, it becomes significant in multilayer structures. Theoretical studies on bilayer Ising superconductors have predicted that the difference in the magnetic vector potential between the two superconducting layers can induce a finite-momentum pairing state, known as the orbital-FFLO state. In this state, Cooper pairs in the two layers acquire equal magnitude but oppositely directed nonzero momenta, reflecting the influence of the magnetic vector potential on the superconducting phase factor under in-plane magnetic fields [55–61]. Recent experimental studies have provided evidence supporting the existence of such finite-momentum pairing states in multilayer Ising superconductors [34,62–70].

In this study, we present a comprehensive analysis of the upper critical field boundary and the superconducting states in layered Ising superconductors, particularly those with weak interlayer Josephson coupling. We identify a novel finite-momentum superconducting configuration that is fundamentally different from those reported in previous studies [59,65]. Our theory demonstrates that the formation and melting of the Josephson vortex lattice are key to understanding the first-order phase transition observed in $\mathrm{Ba_{6}Ta_{11}S_{28}}$ superlattices [70]. The calculated upper critical field and melting line together provide a robust qualitative framework for interpreting experimental observations and offer new insight into the complex phase behavior induced by in-plane magnetic fields in these systems.

## RESULTS

### Main results and phase diagram

**Figure 1.**
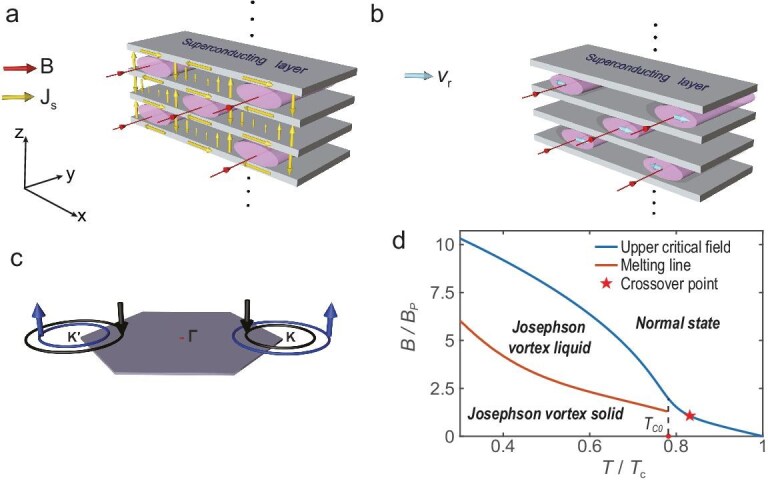
Schematic diagram of the Josephson vortex solid, Josephson vortex liquid and the corresponding phase diagram. (a) Dense Josephson vortex lattice in layered superconductors. The external magnetic field is applied along the *y* direction. The superconducting layers are depicted as gray parallel slabs, while the Josephson vortices are represented by pink elliptical cylinders. Red arrowed lines indicate magnetic flux lines, and yellow arrows illustrate the superconducting current flow. (b) Josephson vortex liquid phase, in which vortices undergo random motion in the *x* direction, with their velocities indicated by light blue arrows. (c) The Ising spin-orbit coupling induces band splitting between opposite spins, locks the spin orientation perpendicular to the plane and results in spin-valley locking. (d) Phase diagram of layered superconductors, featuring the upper critical field line (blue) and the melting line (orange). The states below and above the melting line correspond to the configurations illustrated in panels (a) and (b), respectively. The red pentagram marks the crossover point at which the upper critical field exhibits an upturn. The calculated melting line terminates at the single-layer transition temperature $T=T_{{\rm c}0}$, beyond which the vortex-core size exceeds the interlayer spacing, rendering the term Josephson vortices inapplicable.

We propose that the experimentally observed first-order phase transition in bulk layered superconductors arises from the melting of the Josephson vortex solid into a Josephson vortex liquid [70]. Panels (a) and (b) of Fig. 1 illustrate the distinct states of the Josephson vortices: in the solid phase, the vortices form a static, stretched triangular lattice, as shown in Fig. 1a, whereas in the liquid phase, the vortices can move randomly, primarily along the *x* direction, as depicted in Fig. 1b. Figure 1c illustrates that the electron spins are locked in the out-of-plane directions near the $\mathrm{K}$ and $\mathrm{K}^{\prime }$ valleys, providing robust protection for Cooper pairs against in-plane magnetic fields. Figure 1d presents the phase diagram of the system. The blue line denotes the in-plane upper critical field; beyond this boundary, the order parameter vanishes, signaling a transition from the superconducting state to the normal state. Near the superconducting transition temperature $T_{\rm c}$, the upper critical field displays a linear dependence on temperature, but exhibits an upturn as the temperature approaches the crossover point, marked by a red pentagram. Meanwhile, the orange line shows the calculated melting line, which displays a linear trend at higher temperatures.

### Model

In layered Ising superconductors, the weak Josephson coupling between adjacent layers gives rise to strong anisotropy. The superconducting order parameter is discrete along the out-of-plane direction, which fundamentally distinguishes these systems from conventional bulk superconductors described by the continuous three-dimensional Ginzburg–Landau model. Strong Ising spin-orbit coupling locks electron spins in the out-of-plane direction, effectively suppressing the Zeeman effect induced by the in-plane magnetic fields [52]. As a result, the spin degree of freedom of Cooper pairs can be neglected in our analysis. Layered Ising superconductors can thus be described by the well-known Lawrence–Doniach model, with the total free energy given by [6,71]


(1) \begin{eqnarray*}
F &=& \frac{H_{c}^2(T)}{4\pi } \sum _{l} \int d^2 \mathbf {r} \bigg \lbrace \int ^{lD+d/2}_{lD-d/2} dz \\
&& \times \, \bigg [ \xi _{ab,0}^{2} \left| \left( \mathbf {\nabla }_{\parallel } -i \frac{2\pi }{\Phi _{0}} \mathbf {A}_{\parallel }(\mathbf {r},z) \right) \Psi _{l}(\mathbf {r},z) \right| ^2 \\
&& +\, \xi _{c,0}^{2} \bigg | \bigg ( \frac{\partial }{\partial z} -i \frac{2\pi }{\Phi _{0}} A_{z}(\mathbf {r},z) \bigg ) \Psi _{l}(\mathbf {r},z) \bigg | ^2 \\
&&-\, \big | \Psi _{l}(\mathbf {r},z) \big | ^2 + \frac{1}{2} \big | \Psi _{l}(\mathbf {r},z) \big | ^4 \bigg ] \\
&& -\, \frac{\xi _{ab,0}^{2}}{\lambda _{J}^2} d [ \Psi ^{*}_{l} ( \mathbf {r} ) \Psi _{l+1} ( \mathbf {r} ) e^{-i \chi _{l,l+1}(\mathbf {r})} +{\rm c.c.} ] \bigg \rbrace \\
&& +\, \int d^3 \mathbf {r} \frac{B^2(\mathbf {r},z)}{8 \pi }.
\end{eqnarray*}

In the above equation, we introduce a characteristic scale for the critical field, given by $H_c = {\Phi _0}/{(2\sqrt{2}\pi \xi _{ab,0} \lambda _{ab,0})}$, where $\xi _{ab,0}$, and $\lambda _{ab,0}$ denote the in-plane coherence length and in-plane penetration depth of a single superconducting layer, respectively, and $\xi _{{c},0}$ denotes the single-layer out-of-plane coherence length. These lengths follow a temperature dependence $L = L(0)(1 - {T}/{T_{{\rm c}0}})^{-1/2}$. The subscript ‘$0$’ is used to distinguish these intrinsic single-layer lengths from the corresponding lengths of the bulk system. The parameters *d* and *D* refer to the single-layer thickness and the interlayer spacing, respectively. The Josephson length $\lambda _{J}$, which characterizes the spatial extent of a Josephson vortex in the in-plane direction, is defined as $\lambda _{J}=\gamma D$. The quantity $\chi _{l,l+1}(\mathbf {r})$ is the Peierls phase and is given by $\chi _{l,l+1}(\mathbf {r}) =({2\pi }/{\Phi _{0}}) \int _{lD}^{(l+1)D} dz A_{z}(\mathbf {r},z)$, where $A_{z}$ is the out-of-plane component of the magnetic vector potential, while $A_{\parallel }$ represents its in-plane component. The order parameter $\Psi _{l}$ for the $l$th layer is normalized by $\Psi _{0} = \sqrt{ -{\alpha }/{\beta }}$, with $\alpha$ and $\beta$ being the coefficients of the second- and fourth-order terms, respectively, in the Ginzburg–Landau free-energy expansion.

### Upper critical field of the bulk layered Ising superconductors

Near the upper critical field, the magnetic field is assumed to penetrate the superconductor uniformly along the $y$ direction, i.e. $\mathbf {B} = B \mathbf {\hat{y}}$. We adopt a gauge in which the magnetic vector potential has only an $x$ component, $\mathbf {A} = Bz \mathbf {\hat{x}}$, and $A_{z} = 0$. This choice leads to vanishing Peierls phases, $\chi _{l,l+1} = 0$. To minimize the free energy, $\Psi _{l}(\mathbf {r},z)$ must be independent of the $y$ and $z$ coordinates. For bulk materials, boundary effects can be neglected, so the modulus of the order parameter in all layers can be described by a single function $f$. Thus, the order parameter takes the form $\Psi _{l}(x) = f(x)e^{i\varphi _{l}(x)}$, where $\varphi _{l}$ is the phase of the order parameter in the $l$th layer. By substituting the above expressions for $\mathbf {A}$ and $\Psi _{l}$ into Equation (1), and neglecting the magnetic energy term independent of the order parameter, we obtain the superconducting-related free energy $F_{s}$:


(2) \begin{eqnarray*}
F_{s}&=& \frac{H_{c}^2}{4\pi } \sum _{l} \int d^2 \mathbf {r} \bigg \lbrace \int ^{lD+d/2}_{lD-d/2} dz \bigg [ \xi _{ab,0}^{2} \bigg ( \bigg | \frac{df}{d x} \bigg |^2 \\
&& +\, \bigg | \bigg ( \frac{d \varphi _{l}}{d x}-\frac{2\pi }{\Phi _{0}} Bz \bigg ) f \bigg |^2 \bigg )- |f | ^2 + \frac{1}{2} | f | ^4 \bigg ] \\
&&-\, 2 \displaystyle\frac{\xi _{ab,0}^{2}}{\lambda _{J}^2}d \, \cos (\varphi _{l+1}-\varphi _{l}) |f | ^2 \bigg \rbrace .
\end{eqnarray*}

We then assume that $\varphi _{l} = Q_{l}x$, where $Q_l$ is the Cooper-pair momentum in the $l$th layer. According to Equation (2), if $Q_{l}$ is uniform across all layers (with the uniform superconducting state corresponding to the special case $Q_{l} = 0$), similar to the FF state discussed in previous studies [58–60], the in-plane kinetic energy term grows rapidly with layer index. To further lower the free energy by minimizing the term $({d \varphi _{l}}/{dx} - {2\pi Bz}/{\Phi _{0}} )^2$ in Equation (2), we propose that $Q_l$ varies with the layer index. Specifically, $Q_{l} = {2\pi B z_{l}}/{\Phi _{0}}$, where $z_{l}$ is an undetermined constant. Minimizing the free energy with respect to $z_{l}$ yields $z_{l} = lD$, which corresponds to the $z$ coordinate of the centre of the *l*th layer. A detailed discussion of the physical justification for this momentum configuration is provided in the Section S2. This configuration extends the finite-momentum pairing proposed in bilayer TMD systems [55]. We refer to this exotic state, in which the momentum difference of Cooper pairs between adjacent layers is fixed ($\Delta Q={2\pi BD}/{\Phi _{0}}$) as the orbital-FFLO state. Minimizing the free energy with respect to $f$ in the absence of a magnetic field establishes a quantitative relationship between the transition temperature of an individual layer and that of the whole system, $T_{\rm c} = ( {2 \xi _{ab,0}^2(0)}/{\lambda _{J}^2} + 1 ) T_{{\rm c}0}$, which indicates that interlayer Josephson coupling enhances the transition temperature of the system.

Substituting the form of $\varphi _{l}$ into Equation (2) and performing the spatial integration, we arrive at the following free-energy density:


(3) \begin{eqnarray*}
f_{s}&=& \frac{H_{c}^2}{4\pi } \sum _{l} \int _{0}^{1} d\bar{x} \bigg \lbrace \frac{\xi _{ab,0}^{2}}{L_{0}^2} \bigg ( \bigg | \frac{df}{d \bar{x}} \bigg |^2 + \frac{\pi ^2}{3} \epsilon ^2 |f | ^2 \bigg ) \\
&&-\, |f | ^2 + \frac{1}{2} | f | ^4 - 2\frac{\xi _{ab,0}^{2}}{\lambda _{J}^2} \, \cos (2\pi \bar{x} ) |f | ^2 \bigg \rbrace . \\
\end{eqnarray*}

The free-energy density is defined as $f_{s}={F_s}/{Sd}$, where *S* denotes the area of the superconducting layers. The characteristic length scale $L_{0} = {\Phi _{0}}/{(BD)}$ and the dimensionless coordinate $\overline{x} = {x}/{L_{0}}$ are introduced in the above expression. In fact, the cosine term $\cos (2\pi \overline{x})$ in the Josephson coupling requires that the modulus of the order parameter also has a period of 1. Therefore, to obtain the free-energy density of the system, it is sufficient to integrate over a single period, as shown in Equation (3). Here, the term ${\pi ^2}\epsilon ^2 |f|^2 /{3}$ in the bracket represents the diamagnetic energy arising from the finite thickness $d$, and the parameter $\epsilon$ is defined as the ratio of the superconducting-layer thickness to the interlayer spacing, $\epsilon =d/D$. The superconducting phase boundary is then determined numerically by solving an eigenvalue problem (see Section S1).

**Figure 2.**
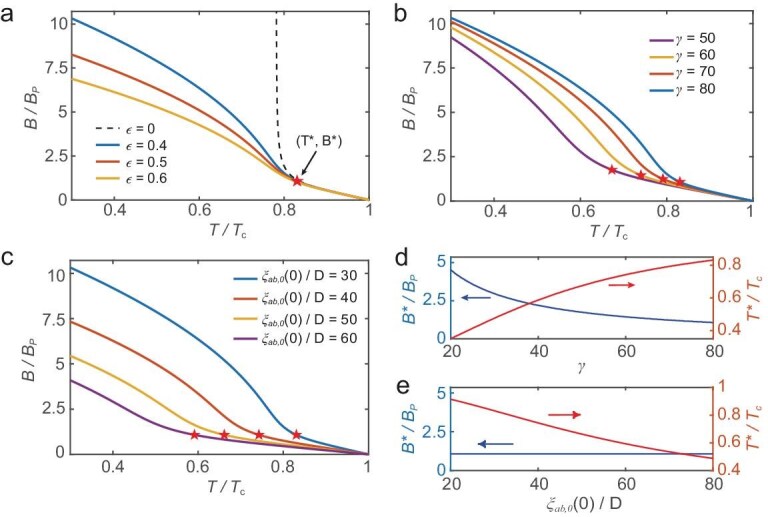
In-plane upper critical fields of bulk layered superconductors: parameter dependence and crossover behavior. (a) In-plane upper critical fields under varying ratios $\epsilon$, with the anisotropy fixed at $\gamma = 80$ and the in-plane coherence length at $\xi _{ab,0}(0)/D = 30$. (b) In-plane upper critical fields as a function of anisotropy $\gamma$, with the in-plane coherence length fixed at $\xi _{ab,0}(0)/D = 30$ and the ratio $\epsilon = 0.4$. (c) In-plane upper critical fields for various in-plane coherence lengths, under fixed conditions $\gamma = 80$ and $\epsilon = 0.4$. Crossover points are marked by red pentagrams. (d) Magnetic-field and temperature values of the crossover point as functions of the anisotropy $\gamma$, with $\xi _{ab,0}(0)/D = 30$ and $\epsilon = 0.4$ fixed. (e) Magnetic-field and temperature values at the crossover point as functions of in-plane coherence length $\xi _{ab,0}(0)/D$, with $\gamma = 80$ and $\epsilon = 0.4$ fixed. Throughout all panels, the transition temperature is fixed at $T_{\rm c} = 2.7 \, \mathrm{K}$. The Pauli-limit field $B_{P}$ is used as the magnetic-field scale, following the relation $B_{P}=1.86 \, T_{\rm c}$.

Figure 2 illustrates the dependence of the upper critical field on various parameters and the crossover from three- to two-dimensional behavior in layered systems. Figure 2a shows that, when the layer thickness is zero ($\epsilon = 0$), the upper critical field diverges near the crossover point. This anomaly disappears for finite $\epsilon$ values [71]. Increasing the thickness of an individual superconducting layer significantly reduces the upper critical field, owing to the enhanced diamagnetic energy term ${\pi ^2}\epsilon ^2 |f|^2/{3}$. Panels (b) and (c) of Fig. 2 show that both anisotropy $\gamma$ and the zero-temperature in-plane coherence length $\xi _{ab,0}(0)$ affect the upper critical field. We extract the magnetic field and temperature at the crossover point and analyze their dependence on $\gamma$ and $\xi _{ab,0}(0)$, as shown in Fig. 2d and e. These results can be understood as follows. When the out-of-plane coherence length $\xi _{c}(T)\gg D$, the system exhibits three-dimensional superconducting coherence. In contrast, for $\xi _{c}(T)< D$, interlayer coherence is weak and the system behaves more two dimensionally. At the crossover point, $\xi _{c}(T^{*})\simeq D$. According to the anisotropic relation $\xi _{c}={\xi _{ab}}/{\gamma }$, $\xi _{c}(0)$ increases with larger $\xi _{ab,0}(0)$ (as $\xi _{ab}(0)$ is positively correlated with $\xi _{ab,0}(0)$) and smaller $\gamma$, leading to a lower crossover temperature $T^{*}$. For three-dimensional superconductors, the in-plane upper critical field is given by $H_{c2}^{||}(T)= {\Phi _{0}}/{[2\pi \xi _{ab}(T) \xi _{c}(T)]}$. At the crossover point, where $\xi _{c}(T^{*})\simeq D$, the upper critical field simplifies to $H_{c2}^{||}(T^{*})\simeq {\Phi _{0}}/{(2\pi \gamma D^2)}$, indicating that the crossover magnetic field is inversely proportional to the anisotropy $\gamma$ and independent of $\xi _{ab,0}(0)$. The dimensional crossover point has been observed experimentally and shown to be tunable by adjusting the interlayer Josephson coupling, either through tuning chemical intercalation of various guest species [72,73] or by employing double-side ionic-gating techniques [74].

It should be emphasized that these calculations are specifically applicable to bulk layered Ising superconductors. Systems consisting of only a few layers exhibit substantially different behavior [55–60]. In such cases, alternative Cooper-pair momentum configurations may arise, potentially leading to more energetically favorable states in certain regions of the phase diagram. A systematic comparison of superconductors with different layer numbers is provided in Section S2.

### Characteristic of the Josephson vortex lattice

This dimensional crossover can also be understood from the perspective of vortices. When the temperature approaches the system transition temperature $T_{\rm c}$, vortex cores span multiple layers, and the destruction of superconductivity by the magnetic field displays bulk-like behavior. As demonstrated in Section S3, when the temperature decreases to the single-layer transition temperature $T_{\rm {c}0}$, the vortex-core size becomes comparable to the interlayer spacing, confining the vortices to the interlayer regions. These vortices are then referred to as Josephson vortices [3–9]. As the magnetic field increases, Josephson vortices arrange into a stretched triangular lattice. In this section, we discuss the formation and properties of the Josephson vortex lattice.

**Figure 3.**
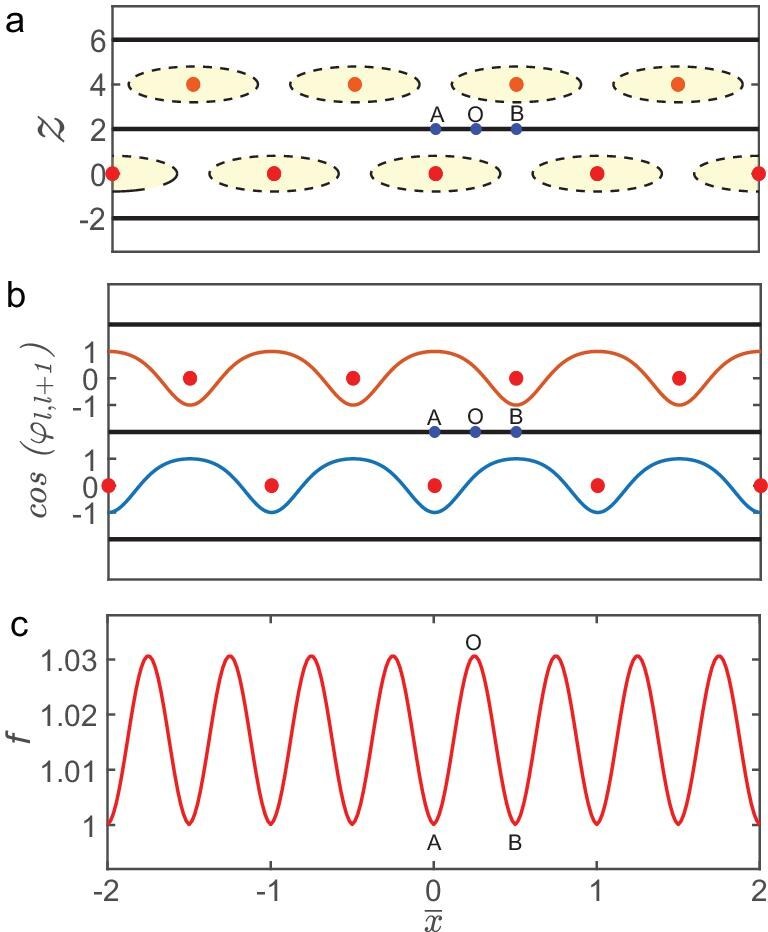
Phase and modulus behavior of the order parameter in the presence of the Josephson vortex lattice. (a) Planar view of the Josephson vortex lattice observed along the magnetic-field direction. The black planes represent superconducting layers, yellow ovals indicate Josephson vortex cores and red points mark the centers of these cores. The points *A, O* and *B* correspond to the coordinates $\bar{x}=0$, $\bar{x}=\frac{1}{4}$ and $\bar{x}=\frac{1}{2}$, respectively. (b) Spatial variation of the cosine of the phase difference between neighboring superconducting layers. The blue curve shows the phase difference between even *l* and odd $l+1$ layers, while the orange curve shows the phase difference between odd *l* and even $l+1$ layers, as calculated from Equation (5). (c) Spatial variation of the modulus of the order parameter. The point *O* corresponds to the maximum value, while points *A* and *B* correspond to the minimum values. The periodicity of the modulus is half that of the Josephson vortex lattice. The parameters used are $T_{\rm c}=2.7 \, \mathrm{K}$, $D=1.2 \, \mathrm{nm}$, $\epsilon =0.4$, $\gamma = 80$, $\xi _{ab,0}(0)/D = 30$. All panels are calculated at $B = 1.8 B_{P}$ and $T = 0.6 \, T_{\rm c}$.

Here, we focus on the case of a densely packed Josephson vortex lattice, as vortex-lattice melting is most likely to occur in such configurations. The in-plane period of the Josephson vortex is given by $L_{0} = \frac{\Phi _{0}}{BD}$. A planar view of the Josephson vortex lattice observed along the magnetic-field direction is shown in Fig. 3a. The actual magnetic field within the superconductor is non-uniform and can be written as $B_v = B + \Delta B(x)$, where *B* is the applied magnetic field and $\Delta B$ is a small periodical modulation. We decompose the free energy in Equation (2) into two parts: $F_{s,\varphi }$, which is associated with the phase $\varphi _{l}$, and $F_{s,f}$, which depends only on the modulus of the order parameter *f*. The properties of the Josephson vortex lattice are determined by the phase-dependent free energy $F_{s,\varphi }$. Owing to the periodic structure introduced by the Josephson vortex lattice, the Josephson current $J_{s} \propto \sin (\varphi _{l,l+1})$ must exhibit the same structural characteristics. Since the pure phase form of the orbital-FFLO state is inadequate, we assume that the phase $\varphi _{l}$ takes the form $\varphi _{l} = Q_{l}x + P_{l}(x) + C_{l}$, where $Q_{l}$ is the finite momentum of the orbital-FFLO state discussed previously, $P_{l}(x)$ is a small periodic oscillation along the *x* direction and $C_{l}$ is a layer-dependent constant. Then $F_{s,\varphi }$ can be written as


(4) \begin{eqnarray*}
F_{s,\varphi }&=& \frac{H_{c}^2 d}{4\pi } \sum _{l} \int d^2 \mathbf {r} \bigg \lbrace | f |^2 \bigg [ \xi _{ab,0}^2 \bigg | \frac{d P_{l}}{d x} \bigg |^2 \\
&&-\, \frac{\xi _{ab,0}^2}{\lambda _{J}^{2}} \, \cos ( \varphi _{l+1}- \varphi _{l} ) \bigg ] \bigg \rbrace .
\end{eqnarray*}

The layer-dependent phase factor of the Josephson vortex lattice can be obtained analytically as


(5a) \begin{eqnarray*}
\varphi _{l}(x)=Q_{l}x+S_{l}\pi +P_{l}(x),
\end{eqnarray*}

(5b) \begin{eqnarray*}
\quad S_{l} &=& \left\lbrace
\begin{array}{@{}l@{\quad }l@{}}l/{2} & \text{if } l \text{ is even}, \\
&{(l+1)}/{2}\ \text{if } l \text{ is odd}. \end{array}\right.
\end{eqnarray*}

The term $S_{l}\pi$ arises from the phase change between neighboring layers caused by the formation of the Josephson vortex lattice. The oscillation term is given by


(6) \begin{eqnarray*}
P_{l}(x) = (-1)^{l}\frac{L_{0}^2}{2\pi ^2 \lambda _{J}^2} \mathrm{sin} \bigg ( { \frac{2\pi x}{L_{0}}} \bigg ),
\end{eqnarray*}

(see Section S4 for details). Substituting $\varphi _{l}$ back into Equation (4) and introducing the dimensionless coordinate $\bar{x} = \frac{x}{L_{0}}$, we derive the complete expression for the free-energy density in the presence of a densely packed Josephson vortex lattice,


(7) \begin{eqnarray*}
f_{s}&=& \frac{H_{c}^2}{4\pi } \sum _{l} \int _{0}^{1} d\bar{x} \bigg \lbrace \frac{\xi _{ab,0}^2}{L_{0}^2} \bigg ( \bigg | \frac{d f}{d \bar{x}} \bigg |^2 + \frac{\pi ^2\epsilon ^2}{3} |f | ^2 \bigg ) \\
&&\quad - |f | ^2 + \frac{1}{2} |f | ^4 \\
&&\quad +\bigg ( \frac{\xi _{ab,0} L_{0}}{\pi \lambda _{J}^2} \bigg ) ^2 \bigg [ \frac{3}{2} \cos (4 \pi \overline{x}) - \frac{1}{2} \bigg ] |f | ^2 \bigg \rbrace . \\
\end{eqnarray*}

Figure 3b shows the cosine of the phase differences between neighboring superconducting layers. Minimization of Equation (7) reveals the spatial variation of the modulus of the order parameter, with the results presented in Fig. 3c. The modulus exhibits periodic oscillations with a period of $L_{0}/2$, where $L_{0}$ is the in-plane period of the Josephson vortex lattice. This behaviour can be understood intuitively as a consequence of both vortex-induced suppression and the underlying spatial symmetry. The symmetry of the lattice ensures that points *A*($\overline{x}=0$) and *B*($\overline{x}=\frac{1}{2}$) experience identical suppression from neighboring vortices, resulting in $f(0)=f(\frac{1}{2})$. This equality can be generalized to $f(\overline{x}_{0})=f(\overline{x}_{0}+\frac{1}{2})$, thereby halving the period of the modulus. Moreover, the inversion center at point *O* ($\overline{x}=\frac{1}{4}$) is maximally distant from the vortex cores and experiences the weakest suppression, resulting in the maximum modulus value, whereas points *A* and *B* correspond to the minimum modulus values, as illustrated in Fig. 3c. Unlike Abrikosov vortices, which strongly suppress the superconducting order parameter near their cores, Josephson vortices exerts a significantly weaker perturbation. As a result, the spatial variation of the modulus is weak, amounting to only about 1.5% of its mean value at $B = 1.8 B_{\rm P}$ and $T = 0.6 \, T_{\rm c}$. The presence of Josephson vortices and their influence on the phase configuration are consistent with the recent results obtained using a microscopic model in bilayer Ising superconductors [75].

### Melting of the Josephson vortex lattice

A physical interpretation of the first-order transition in the superconducting phase is as follows. Below the transition line, magnetic vortices are arranged in an ordered lattice. At the transition, the Josephson vortex lattice melts into a Josephson vortex liquid, in which vortices exhibit random microscopic motion and the magnetic field becomes more uniform. In the following, we derive the first-order melting line and demonstrate that this melting transition can provide a quantitative explanation for the first-order phase-transition features observed in a recent experiment [70].

At finite temperatures, thermal fluctuations cause vortices to vibrate around their equilibrium positions. These deviations are governed by the elastic moduli of the Josephson vortex lattice, which characterize lattice’s resistance to deformation and are crucial for understanding its thermal behavior. The energy variation due to deformation of the Josephson vortex lattice is given by


(8) \begin{eqnarray*}
\Delta F &=& \frac{1}{2} \sum _{k} [c_{11} k_{x}^{2}+c_{44} k_{y}^{2}+c_{66}\tilde{k}_{z}^{2} ] |U_{k} |^2, \\
c_{11} &=& c_{44}=\frac{B^{2} \epsilon }{4\pi \lambda _{ab,0}^{2} \tilde{k}_{z}^{2}} | \overline{f} |^2, \\
c_{66} &=& \frac{\Phi _{0}^2 \epsilon }{32 \pi ^3 \gamma ^{4} \lambda _{ab,0}^{2} D^{2}} | \overline{f} |^2 .
\end{eqnarray*}

Here, $c_{11}$ is the uniaxial compression modulus along the *x* direction, $c_{44}$ is the tilt modulus in the *x*-*y* plane and $c_{66}$ is the shear modulus in the *x*-*z* plane [76]. In a dense Josephson vortex lattice, $c_{11}$ and $c_{44}$ are equivalent [3]. The quantity $\overline{f}$ denotes the average modulus of the order parameter, as calculated from Equation (7); $\tilde{k}_{z}$ is the modified wave-vector component along the out-of-plane direction, defined as $\tilde{k}_{z} = 2 \, \sin ({k_{z}D}/{2})/D$. In our calculations of vibrations along the *x* direction, we neglect the additional elastic modulus $c_{16}$, which arises from the reduced symmetry of the Josephson vortex lattice compared with hexagonal lattices such as the Abrikosov vortex lattice. Detailed derivations of the elastic moduli are provided in Section S5.

To determine the melting line of the Josephson vortex lattice, we employ the Lindemann criterion. According to this criterion, melting occurs when the ratio $\langle u^2 \rangle /L_{0}^{2}$ equals $c_{L}^{2}$, where the Lindemann parameter $c_L$ typically ranges from 0.1 to 0.3 [10–16]. We investigate the displacement of the Josephson vortex lattice induced by thermal fluctuations, taking into account the pinning effect in the insulating regions between the superconducting layers [77]. The vibration of the vortices is predominantly along the *x* direction and is denoted by $u_{\mathbf {k}}^{x}$. The total force acting on the vortices can be expressed as $f^{x}_{\mathbf {k}}= [\Phi _{xx}(\mathbf {k}) + \alpha _{L} ] u_{\mathbf {k}}^{x}$, where $\Phi _{xx}(\mathbf {k})$ is the lattice force constant, defined as $\Phi _{xx}(\mathbf {k}) = c_{11}k_{x}^{2} + c_{44}k_{y}^{2} + c_{66} \tilde{k}_{z}^{2}$. The Labusch parameter $\alpha _{L}$ quantifies the restoring force constant due to impurity pinning acting on the vortices [14]. We define $\Phi ^{\prime }_{\mathbf {k}} = \Phi _{xx}(\mathbf {k}) + \alpha _{L}$; its inverse, $(\Phi ^{\prime }_{\mathbf {k}})^{-1}$, corresponds to the response function associated with thermal fluctuations [10–14]. The mean-square displacement $\langle u^2 \rangle$ is then given by


(9) \begin{eqnarray*}
\langle u^2 \rangle &=& k_{B}T \int _{-\infty }^{\infty } \frac{dk_{y}}{\pi } \int _{BZ} \frac{d^2 \mathbf {k}}{(2\pi )^2} \\
&&\times \, \frac{1}{c_{11}k_{x}^{2} + c_{44}k_{y}^{2} + c_{66}\tilde{k}_{z}^{2} + \alpha _{L}}.
\end{eqnarray*}

The unit cell of the dense Josephson vortex lattice is a parallelogram in the *x*-*z* plane, with lattice vectors $\mathbf {a_{1}} = (L_{0},0,0)$ and $\mathbf {a_{2}} = ({L_{0}}/{2},0,D)$. The corresponding reciprocal lattice vectors are $\mathbf {b_{1}} = 2\pi ({1}/{L_{0}},0,-{1}/{2D})$ and $\mathbf {b_{2}} = 2\pi (0, 0, {1}/{D})$. Periodicity is absent in the *y* direction, and the Dirichlet boundary condition constrains $k_y$ to take discrete values $k_{y}={n\pi }/{L_{y}}$, where *n* is an integer. The factor ${1}/{\pi }$ in the $k_{y}$ integral arises from the wave-vector density of $k_{y}$. To express the Labusch parameter $\alpha _{L}$ in dimensionless form, we introduce a stiffness scale $E_{0}$ defined as $E_{0} = {B_{\lambda _{J}}^2}/{(2\pi ^2 \lambda _{0}^{2})}$, where $B_{\lambda _{J}}={\Phi _{0}}/{(\lambda _{J} D)}$. In our calculations, we use $\lambda _{0}/D =1000$.

**Figure 4.**
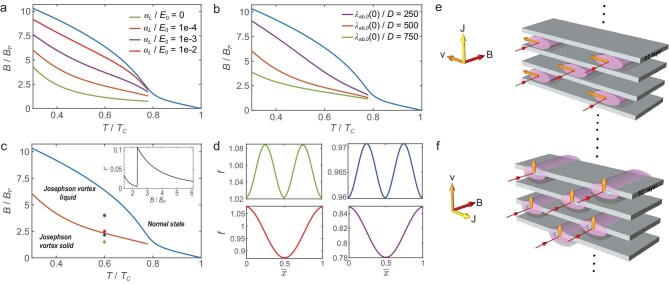
Josephson vortex melting lines and their dynamic characteristics under applied current. (a) Josephson vortex melting line as a function of the pinning strength $\alpha _{L}$, with the in-plane penetration depth fixed at $\lambda _{ab,0}(0)/D = 500$. (b) Josephson vortex melting line as a function of the in-plane penetration depth, with the Labusch parameter fixed at $\alpha _{L}/E_{0} = 1 \times 10^{-4}$. Parameters used are $T_{\rm c} = 2.7\, \mathrm{K}$, $D=1.2 \, \mathrm{nm}$, $\epsilon =0.4$, $\gamma = 80$, $\xi _{ab,0}(0)/D = 30$, $B_{P}=1.86 \, T_{\rm c}$ and the Lindemann parameter $c_{L}=0.1$. (c) Phase diagram exhibiting three distinct regions. The upper critical field line separates the orbital-FFLO state from the normal state, while the melting line distinguishes between the Josephson vortex solid from the Josephson vortex liquid. The Josephson vortex lattice melts at $B/B_{P}=2.34$ when the temperature $T/T_{\rm c}=0.6$. Green and blue points indicate states below the melting line, whereas red and purple points lie above it, corresponding to $B/B_{P} = 1.5, 2.32, 2.36$ and $4.0$, respectively. The inset shows the modulation of the order-parameter modulus (defined as the variation-to-mean ratio of the modulus), as a function of magnetic-field strength, revealing a pronounced discontinuity at the melting transition. The melting line shown corresponds to $\lambda _{ab,0}(0)/D = 500$ and $\alpha _{L}/E_{0} = 1 \times 10^{-4}$. (d) Spatial distributions of the order-parameter modulus at four different magnetic-field strengths corresponding to the points in panel (c). (e and f) Schematic diagrams of Josephson vortex dynamics driven by interlayer (e) and intralayer (f) currents. Yellow and orange arrows denote the directions of the applied current and the resulting vortex motion, respectively. Light blue arrows in panel (f) represent stochastic motion of vortices along the *x* direction.

Figure 4a examines the dependence of the Josephson vortex melting line on the pinning strength $\alpha _L$. Enhanced pinning strength effectively restricts the vibrations of Josephson vortices, shifting the melting line to higher magnetic fields and temperatures. Figure 4b shows that an increased in-plane penetration depth of an individual superconducting layer, $\lambda _{ab,0}(0)$, reduces the elastic moduli, enhances vortex vibrations and consequently lowers the melting line. Note that the melting line terminates at the temperature $T_{{\rm c}0}$, above which the length $\lambda _{ab,0}$ can no longer be well defined. This termination may also be understood as a limitation of the Josephson vortex description, which is applicable only at temperatures below $T_{{\rm c}0}$. We discuss this point in detail in Section S3. Upon melting, vortices move randomly along the *x* direction, allowing the magnetic field to be treated as homogeneous. The spatial variation of the order-parameter modulus is governed by the minimisation of the free energy in Equation (3).

Panels (c) and (d) of Fig. 4 show that, upon crossing the melting line, the spatial variation of the modulus *f* undergoes a sudden increase at $T/T_{\rm c} = 0.6$, which can serve as an experimental signature of Josephson vortex melting. Although our theoretical calculations suggest a simultaneous doubling in the periodicity of the order parameter’s modulus, we do not take into account the short-range superconducting coherence induced by the random motion of Josephson vortices, which can lead to a smeared periodic modulation of the superconducting gap at large scales.

Our theory predicts distinct transport behaviors of Josephson vortices in response to intralayer and interlayer currents after melting. In Fig. 4e, a small interlayer current induces directional motion along the $-x$ direction, resulting in a finite resistance. When a small current is applied along the *x* direction, the vortices move directionally along the *z* direction while simultaneously exhibiting stochastic motion along the *x* axis, as indicated by the orange and light blue arrows in Fig. 4f, respectively. The superconducting layers act as periodic potential barriers, whose height exceeds that of impurity potentials in the in-plane direction, thereby impeding vortex motion. As a result, the intralayer resistance increases more slowly with magnetic field and temperature than the interlayer resistance, which rapidly approaches its normal-state value upon vortex lattice melting, while the intralayer resistance remains suppressed. In Section S7, we investigate the effective barrier height before and after melting using a thermally activated model [78,79]. Our analysis reveals that the barrier height exhibits markedly different behavior in the solid and liquid states.

**Figure 5.**
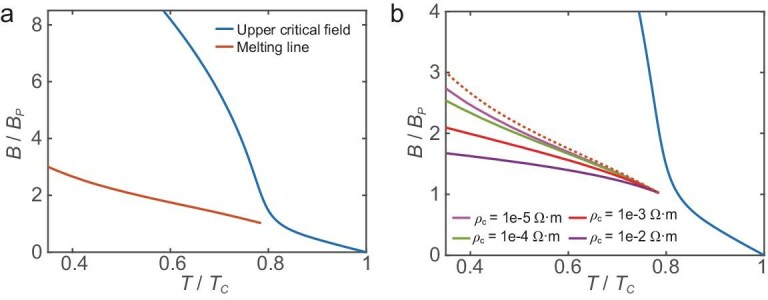
(a) The theoretically calculated upper critical field (blue line) and the melting line induced by thermal fluctuations (orange). (b) Comparison of melting mechanisms driven by thermal fluctuations alone and by combined thermal and quantum fluctuations. The orange dotted line denotes the melting line arising solely from thermal fluctuations, whereas the pink, green, red and purple lines represent melting lines induced by the combined effects of thermal and quantum fluctuations at different values of the resistivity $\rho _{c}$. Parameters used are $T_{\rm c}=2.77 \, \mathrm{K}$, $D = 1.2\, \mathrm{nm}$, $\epsilon =0.25$, $\gamma =100.8$, $\xi _{ab,0}(0)=44.7\, \mathrm{nm}$, $\lambda _{ab,0}(0)/D=700$, $\alpha _{L}/E_{0}=1.8\times 10^{-4}$ and $c_{L}=0.1$. The magnetic-field scale is set to $B_{\rm P}=5 \, \mathrm{T}$.

Figure 5 examines the effects of thermal and quantum fluctuations on Josephson vortex melting. In Fig. 5a, we use an alternative parameter set to illustrate the melting line driven exclusively by thermal fluctuations. Notably, at high temperatures, this melting line exhibits linear behavior, appearing as an extension of the linear segment of the upper critical field. Remarkably, the theoretically calculated melting line arising from thermal fluctuations agrees well with the transition line reported in a recent experimental study of the bulk layered $\mathrm{Ba_{6}Ta_{11}S_{28}}$ system [70]. This superlattice system, $\mathrm{Ba_{6}Ta_{11}S_{28}}$, consists of alternating superconducting and insulating layers, in which electron tunneling between adjacent superconducting $\mathrm{TaS_{2}}$ layers is weak. The strong Ising SOC in the $\mathrm{TaS_{2}}$ layers locks electron spins perpendicular to the in-plane direction, allowing the spin degree of freedom to be neglected under magnetic fields. This behavior is consistent with our theoretical model in Equation (1). Consequently, the upper critical field in this system exhibits a pronounced upward curvature at low magnetic fields, which is quantitatively consistent with the orbital-effect-induced finite-momentum pairing state (orbital-FFLO state).

Figure 5b compares the melting line derived from pure thermal fluctuations with that obtained from the combined effects of thermal and quantum fluctuations, calculated using the fluctuation-dissipation theorem [16] (see Section S6 of the online supplementary material for details). Our results show that quantum effects enhance vortex vibrations, thereby consistently shifting the melting line to lower values relative to that obtained by considering thermal fluctuations alone. Moreover, higher resistivity leads to stronger quantum fluctuations, resulting in a further suppression of the melting line.

## DISCUSSION

Our calculated melting line closely matches the first-order phase-transition line observed in recent experiments on bulk superconducting superlattices. The scenario of Josephson vortex melting transition bridges our theoretical predictions with experimental results, offering a unified perspective on the behavior of layered superconductors under an in-plane magnetic field.

To analyze the superconducting phase boundary between the orbital-FFLO state and the normal state, we propose a novel momentum configuration of Cooper pairs that varies with the layer index. This configuration differs distinctly from previously studied cases, which involve either a uniform momentum across all layers [59] or alternating positive and negative momenta [65]. The proposed finite-momentum pairing state contrasts with the conventional FFLO state: whereas, in the latter, the finite momentum of Cooper pairs originates from Fermi-surface mismatch induced by the Zeeman effect, the finite-momentum pairing state considered here primarily arises from the interplay between the orbital effect of the magnetic field and the layered structure of the superconductor. It is important to note that both the magnetic vector potential and the specific momentum configuration of Cooper pairs are gauge dependent and can change under a gauge transformation. However, analogous to the Aharonov–Bohm effect, the most physically relevant and gauge-invariant quantity is the phase difference between adjacent superconducting layers, which manifests itself in measurable phenomena, such as periodic oscillations in the modulus of the superconducting order parameter.

In the main text, we primarily focus on the phase diagram of the orbital-FFLO state in bulk Ising superconductors. However, few-layer Ising superconductor systems host various superconducting states that differ significantly from their bulk counterparts. We demonstrate the evolution of the phase diagram with layer number and find that the region of orbital-FFLO state expands as the number of layers increases. Although Josephson vortices can form in few-layer superconductors, analyzing their lattice structure and melting transition is challenging owing to boundary effects and the loss of periodicity along the *z* direction. Finally, the present analysis is specifically applicable to layered superconductors with weak Josephson coupling. In systems with strong Josephson coupling, the characteristic upturn of the upper critical field near $T_{c}$ disappears. In such cases, Josephson vortices are replaced by Abrikosov vortices, which lie outside the scope of the present study.

## Supplementary Material

nwag084_Supplemental_File
