# Supplementary material for: Orbital-effect-induced finite-momentum pairing and Josephson vortex lattice melting in layered Ising superconductors
Source: Natl Sci Rev. 2026 Feb 10;13(8):nwag084. doi: 10.1093/nsr/nwag084 (PMC13131243; doi:10.1093/nsr/nwag084)
Supplement: nwag084_Supplemental_File [file nwag084_supplemental_file.pdf]

Orbital effect induced finite-momentum pairing and Josephson vortex lattice melting in layered Ising superconductors

Hongyi Yan[1,2], Haiwen Liu [1,2,3*] Yi Liu[4,5], Ding Zhang[6,7,8,9], X.C. Xie[3,10,11]

[1]Center for Advanced Quantum studies, School of Physics and Astronomy, Beijing Normal University, Beijing 100875, China

[2]Key Laboratory of Multiscale Spin Physics, Beijing Normal University, Beijing 100875, China

[3]Interdisciplinary Center for Theoretical Physics and Information Sciences, Fudan University, Shanghai 200433, China

[4]Department of Physics and Beijing Key Laboratory of Opto-electronic Functional Materials & Micronano Devices, Renmin University of China, Beijing 100872, China

[5]Key Laboratory of Quantum State Construction and Manipulation, Renmin University of China, Beijing 100872, China

[6]State Key Laboratory of Low Dimensional Quantum Physics and Department of Physics, Tsinghua University, Beijing 100084, China

[7]Beijing Academy of Quantum Information Sciences, Beijing 100193, China

[8]Frontier Science Center for Quantum Information, Beijing 100084, China

[9]RIKEN Center for Emergent Matter Science (CEMS), Wako 351-0198, Japan

[10]International Center for Quantum Materials, School of Physics, Peking University, Beijing 100871, China

[11]Hefei National Laboratory, Hefei 230094, China

*Corresponding author. E-mail: haiwen.liu@bnu.edu.cn

CONTENT

Suppl. Note 1. Numerical calculation of upper critical field

Suppl. Note 2. Finite-layer Ising superconductor

Suppl. Note 3. Appearance of Josephson vortex lattice

Suppl. Note 4. Phase fluctuation of dense Josephson vortex lattice

Suppl. Note 5. Elastic moduli of the dense Josephson vortex lattice

Suppl. Note 6. Combined thermal and quantum fluctuations induced melting

Suppl. Note 7. Thermally activated model and effective barrier height

Suppl. Note 1. Numerical calculation of upper critical field

In Eq. (3) of the main text, we derive the free energy density corresponding to the orbital-FFLO state in bulk layered Ising superconductors. By varying the free energy with respect to the modulus f and omitting the fourth-order term, we arrive at the eigenvalue equation for the minimum free energy density:

$$\begin{aligned} \hat{L} f &= E f, \\ \hat{L} &= \frac{d^2}{d\bar{x}^2} + 2\frac{L_0^2}{\lambda_J^2}\cos\left(2\pi\bar{x}\right), \\ E &= \frac{\pi^2}{3}\epsilon^2 - \frac{L_0^2}{\xi_{ab,0}^2(0)}\left(1 - \frac{T}{T_{c0}}\right). \end{aligned} \tag{S1}$$

Here, $\hat{L}$ is a second-order differential operator with respect to $\bar{x}$. The cosine term requires that the eigenstate f must be periodic. We numerically solve this equation by discretizing over one period of length 1, using a sufficiently dense partition (for example, $N = 500$) to ensure accuracy. We then diagonalize the resulting $N \times N$ matrix representation of $\hat{L}$ to obtain its eigenvalues. From Eq. (S1), it is clear that the upper critical field is determined by the largest eigenvalue of $\hat{L}$, which, for a given magnetic field, corresponds to the highest temperature at which superconductivity is completely destroyed.

Suppl. Note 2. Finite-layer Ising superconductor

In the main text, we consider bulk materials with infinite layers, allowing us to ignore boundary effects and assume a uniform modulus of the order parameter across all layers. However, recent experiments have focused on systems with finite layers, ranging from bilayer to several tens of layers [1–3]. As the number of layers decreases, the orbital-FFLO state discussed in the main text may no longer be energetically favorable, suggesting the possibility of alternative momentum configurations for Cooper pairs.

For clarity, we define two distinct states based on their momentum characteristics: 1. the orbital-FFLO state, as previously elucidated in the main text, is characterized by a constant momentum difference q_0 between adjacent superconducting layers. 2. the uniform momentum state, characterized by a uniform momentum q across all layers (including the uniform super-

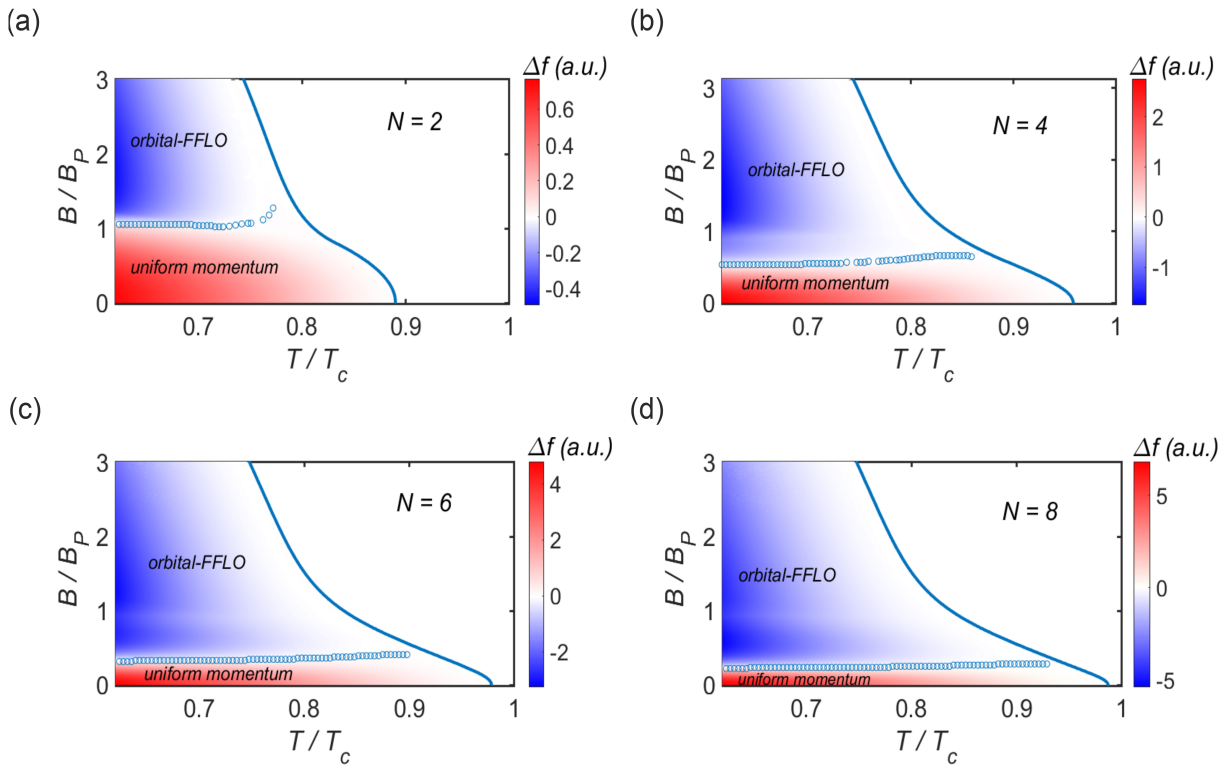

Figure S1. The distribution of the orbital-FFLO state and uniform momentum state in layered superconducting systems with varying numbers of layers. (a) $N = 2$, (b) $N = 4$, (c) $N = 6$ and (d) $N = 8$. Parameter used: $D = 1.2\,\mathrm{nm}$, $\xi_{ab,0}(0)/D = 30$, $\gamma = 80$, $d/D = 0.4$, $T_c = 2.7\,\mathrm{K}$ and $B_P = 1.86\,T_c$. In these diagrams, sky-blue lines mark the upper critical field [4], while sky-blue circles denote first-order phase transition boundaries. The color scale quantifies the free energy difference between the the orbital-FFLO state and the uniform momentum state, $\Delta f = f_{orb} - f_{uni}$: red regions correspond to the thermodynamically stable uniform momentum state ($\Delta f > 0$), whereas blue regions indicate the orbital-FFLO state is favored ($\Delta f < 0$).

conducting state with zero momentum). In this section, we disregard the effects of Josephson vortices and focus solely on these two states. Our aim is to compare their respective regions in the phase diagram and investigate how these regions evolve as the number of layers changes.

For a finite number of layers, the lack of periodicity along the out-of-plane direction leads to a layer-dependent modulus of the order parameter. This necessitates a modification of the free energy expression in Eq. (3) of the main text to adequately account for finite-size effects. We adopt the same gauge as in the main text, $A_x = Bz$, and define the center of the system as the origin of the z-coordinate. Accordingly, for orbital-FFLO state, we assume the order parameter takes the form $\Phi_l(x) = f_l e^{iQ_l x}$ and $Q_l = q_0 \bar{l}$, where the interlayer momentum difference q_0 is treated as a free parameter and $\bar{l} = l - \frac{N+1}{2}$. The free energy density can then be written as follows

$$f_{orb} = \frac{H_c^2}{4\pi} \sum_{l=1}^{N-1} \int_0^1 d\bar{x} \left\{ \frac{\xi_{ab,0}^2}{L_0^2} \left[\left(\frac{q_0 L_0}{2\pi} \right)^2 \left| \frac{df_l}{d\bar{x}} \right|^2 + (q_0 L_0 - 2\pi)^2 \bar{l}^2 |f_l|^2 + \frac{\pi^2}{3} \epsilon^2 |f_l|^2 \right] - |f_l|^2 + \frac{1}{2} |f_l|^4 - 2 \frac{\xi_{ab,0}^2}{\lambda_J^2} \cos(2\pi \bar{x}) f_l f_{l+1} \right\}. \tag{S2}$$

As will be shown below, the product $q_0 L_0$ approaches 2π throughout the entire magnetic field range in the bulk limit, which naturally reduces the above expression to the form of Eq. (3) of the main text.

In the uniform momentum states, the superconducting phase can be uniformly expressed as $\varphi_l = qx$ across all layers. This uniformity eliminates the spatial modulation in the Josephson coupling term presented in Eq. (S2), resulting in a consistent modulus of the order parameter within each layer. Assuming the order parameter takes the form $\Phi_l(x) = f_l e^{iqx}$, the free energy density becomes

$$f_{uni} = \frac{H_c^2}{4\pi} \sum_{l=1}^{N-1} \left\{ \frac{\xi_{ab,0}^2}{L_0^2} \left[\left(qL_0 - 2\pi \bar{l} \right)^2 + \frac{\pi^2}{3} \epsilon^2 \right] |f_l|^2 - |f_l|^2 + \frac{1}{2} |f_l|^4 - 2 \frac{\xi_{ab,0}^2}{\lambda_J^2} f_l f_{l+1} \right\}. \tag{S3}$$

Through optimizing the free energy density, we determine the energetically favorable sets $\{f_l(x), q_0\}$ for the orbital-FFLO state and $\{f_l, q\}$ for the uniform momentum state under specified temperature and magnetic field conditions. We then compare their energies within the phase diagram to assess their relative stability. As shown in Fig. S1, the uniform momentum state dominates the low magnetic field region of the superconducting phase, and the orbital-FFLO state becomes energetically favorable at high magnetic fields. Moreover, as the number of layers increases, the region occupied by the orbital-FFLO state expands. The trend illustrated in Fig. S1 clearly indicates that, as the number of layers approaches infinity, the orbital-FFLO state could potentially occupy the entire superconducting region.

On the other hand, for finite-layer Ising superconductors, the first-order transition line between the orbital-FFLO state and the uniform momentum state, depicted in Fig. S1, accounts for the experimental observations reported in multilayer 2H-$NbSe_2$ systems [3]. In addition,

recent studies on bilayer Ising superconductors [5] have presented phase diagrams consistent with our findings for finite-layer Ising superconductors, albeit with slightly different terminology. Specifically, the orbital-LO state in Ref. [5] corresponds directly to the orbital-FFLO state in our study.

Moreover, we reveal that the phase diagrams for finite layers presented in Fig. S1 at $T > T_{c0}$ correspond to region I of the phase diagram for bulk Ising layer superconductors shown in Fig. S2. In this region, the vortex core size exceeds the interlayer spacing D (See next section), making the use of the uniform magnetic field approximation and the neglect of the Josephson vortex lattice reasonable. The bulk system can be regarded as a special case in which the first-order phase transition extends to zero magnetic field. Notably, the phase diagram in Fig. S2(a) demonstrates broad applicability, capturing the first-order phase transition in both bulk and multilayer systems across different regions of the phase diagram. We further anticipate that the melting of the Josephson vortex lattice will also occur in finite-layer systems at temperature below T_{c0} and under high magnetic fields. We leave this topic for future studies.

In Fig. S3, we present calculations of the interlayer momentum difference q_0 as a function of $\frac{2\pi}{L_0}$ at $T/T_c = 0.7$, where $\frac{2\pi}{L_0}$ is proportional to the magnetic field since $L_0 = \frac{\Phi_0}{BD}$. Our results show that the product $q_0 L_0$ approaches 2π at much lower magnetic fields as the number of layers increases. Therefore, it is reasonable to extrapolate that, in the limit of infinite layers, $q_0 L_0$ equals 2π as soon as the in-plane magnetic field is applied. This behavior is consistent with the results shown in Fig. S1, and further supports the ansatz for the form of the Cooper pair momentum in the orbital-FFLO state, namely $Q_l = \frac{2\pi BlD}{\Phi_0}$, in the bulk limit.

Additionally, our numerical results indicate the presence of small jumps in q_0, in addition to the initial jump from zero to a finite value at lower magnetic field, which we also interpret as first-order phase transitions. However, the experimental signatures of these smaller jumps may not be as pronounced as the zero-to-finite q_0 transitions and could be smeared out by impurities. Moreover, the “few steps” shown in Fig. S3 is in fact a universal feature of multilayer systems. As the number of layers tends to infinity, these small steps becomes negligible. In the bulk limit, we can therefore generalize our result and conclude that the product $q_0 L_0$ approaches 2π over the entire magnetic field range.

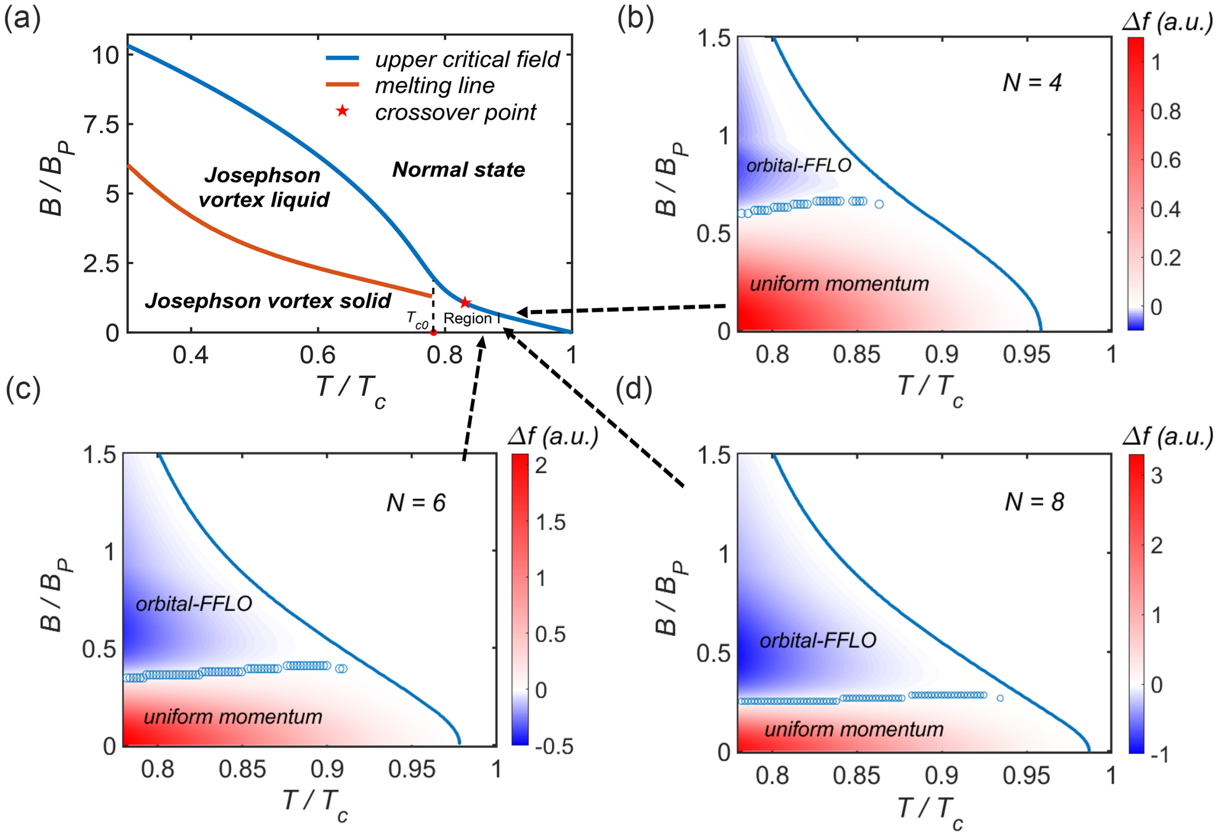

Figure S2. Phase diagrams for systems with different layer number. (a) Phase diagram of bulk layered Ising superconductors. (b-d) Phase diagrams for systems with layer number $N = 4$ (b), $N = 6$ (c) and $N = 8$ (d). Parameter used: $D = 1.2\,\mathrm{nm}$, $\xi_{ab,0}(0)/D = 30$, $\gamma = 80$, $d/D = 0.4$, $T_c = 2.7\,\mathrm{K}$ and $B_P = 1.86\,T_c$. These panels are reproduced from Fig. 1(d) and Fig. S1, respectively, and effectively account for the first-order phase transition observed in the recent experiment [3].

Suppl. Note 3. Appearance of Josephson vortex lattice

In the main text, we conclude that the Josephson vortex lattice emerges at the temperature $T < T_{c0}$, where T_{c0} is the single-layer transition temperature. This conclusion is verified as follows: When the vertical coherence length of the system, $\xi_c(T_J)$, satisfies $\xi_c(T_J) = \frac{D}{\sqrt{2}}$, the vortex core size matches the interlayer spacing D. These vortices are then referred to as Josephson vortices, we have

$$\xi_c(0)\left(1 - \frac{T_J}{T_c}\right)^{-\frac{1}{2}} = \frac{D}{\sqrt{2}}. \tag{S4}$$

Solving for T_J, we obtain

$$\frac{T_J}{T_c} = 1 - \frac{2\xi_c^2(0)}{D^2} = 1 - \frac{2\xi_{ab}^2(0)}{\gamma^2 D^2} = 1 - \frac{2\xi_{ab}^2(0)}{\lambda_J^2}, \tag{S5}$$

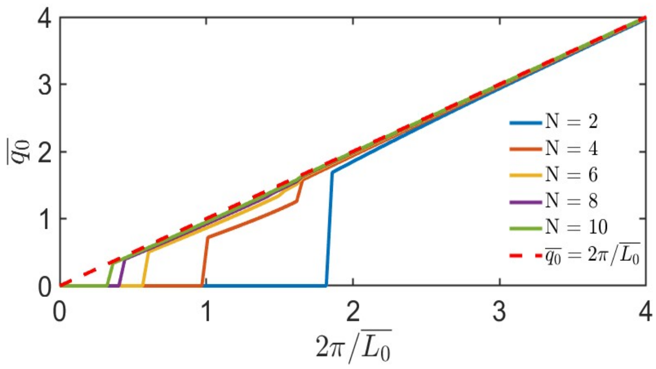

Figure S3. Dimensionless interlayer momentum difference $\overline{q_0}$ as a function of $2\pi/\overline{L_0}$ for layer number of $N = 2, 4, 6, 8, 10$, where $\overline{q_0} = q_0\lambda_J$ and $\overline{L_0} = L_0/\lambda_J$. The red dashed line corresponds to $q_0 L_0 = 2\pi$ in Eq. (S2). The temperature is fixed at $T/T_c = 0.7$.

where we have used the anisotropy relation $\xi_{ab} = \gamma\xi_c$. Next, we substitute the system's in-plane coherence length $\xi_{ab}(0)$ with that of the single-layer $\xi_{ab,0}(0)$, given by

$$\xi_{ab,0}(0) = \sqrt{\frac{\hbar^2}{2M_{||}\alpha_0 T_{c0}}}, \tag{S6}$$

whereas for that of the system, we have

$$\xi_{ab}(0) = \sqrt{\frac{\hbar^2}{2M_{||}\alpha_0 T_c}}. \tag{S7}$$

Therefore, the zero-temperature in-plane coherence lengths satisfy $\xi_{ab}(0) = \sqrt{\frac{T_{c0}}{T_c}}\xi_{ab,0}(0)$. Substituting this into Eq. (S5) yields

$$T_J = T_c - \frac{2\xi_{ab,0}^2(0)}{\lambda_J^2} T_{c0} = T_{c0}, \tag{S8}$$

where we have used the relation $T_c = \left(\frac{2\xi_{ab,0}^2(0)}{\lambda_J^2} + 1\right) T_{c0}$, as derived in the main text. This confirms that the Josephson vortex lattice forms only below T_{c0}, and the melting line is well-defined up to this temperature, above which the elastic moduli in Eq. (7) of the main text cannot be defined.

The Josephson vortex lattice picture does not extend to the upper critical field regime. Instead, its applicability is strictly confined to temperatures below the single-layer transition temperature T_{c0}. Beyond this threshold ($T_{c0} < T < T_c$), the system undergoes a dimension crossover:

the vortex core expands to span multiple layers, causing the system to behave more like a bulk superconductor. This bulk-like phase continuously connects to the upper critical boundary $H_{c2}(T)$, where the linear behavior is characteristic of bulk superconductivity.

Suppl. Note 4. Phase fluctuation of dense Josephson vortex lattice

In the presence of Josephson vortex lattice, the periodicity of the lattice can significantly affects the phase of the superconducting order parameter, causing the phase to deviate from that of the orbital-FFLO state. We decompose the free energy in Eq. (2) of the main text into two components, $F_{s,\varphi}$, associated with the phase φ_l, and $F_{s,f}$, depending solely on the modulus of the order parameter f, as shown below

$$F_{s,\varphi} = \frac{H_c^2}{4\pi} \sum_l \int d^2\mathbf{r} \left\{ \int_{lD-d/2}^{lD+d/2} dz\, \xi_{ab,0}^2 \left| \left(\frac{d\varphi_l}{dx} - \frac{2\pi}{\Phi_0} Bz \right) f \right|^2 - 2\frac{\xi_{ab,0}^2}{\lambda_J^2} d \cos(\varphi_{l+1} - \varphi_l) |f|^2 \right\}, \tag{S9}$$

$$F_{s,f} = \frac{H_c^2 d}{4\pi} \sum_l \int d^2\mathbf{r} \left\{ \xi_{ab,0}^2 \left| \frac{df}{dx} \right|^2 - |f|^2 + \frac{1}{2} |f|^4 \right\}. \tag{S10}$$

Substituting $\varphi_l = Q_l x + P_l(x) + C_l$ into $F_{s,\varphi}$ in Eq. (S9), we obtain

$$F_{s,\varphi} = \frac{H_c^2 d}{4\pi} \sum_l \int d^2\mathbf{r} \left\{ |f|^2 \left[\xi_{ab,0}^2 \left| \frac{dP_l}{dx} \right|^2 - 2\frac{\xi_{ab,0}^2}{\lambda_J^2} \cos\left(\frac{2\pi x}{L_0} + P_{l,l+1} + C_{l,l+1} \right) \right] \right\}. \tag{S11}$$

In the equation above, we define the phase difference between neighboring layers l and $l+1$ as $P_{l,l+1}(x) = P_{l+1}(x) - P_l(x)$, and the difference in the constant part as $C_{l,l+1} = C_{l+1} - C_l$. The total phase difference is then given by $\varphi_{l,l+1}(x) = \frac{2\pi x}{L_0} + P_{l,l+1}(x) + C_{l,l+1}$. The diamagnetic energy term $\frac{\pi^2 \epsilon^2 |f|^2}{3}$, which arises from the finite thickness of the superconducting layers, is phase-independent and can be absorbed into $F_{s,f}$. The Josephson current density $\mathbf{J} = -c\frac{\delta F}{\delta \mathbf{A}}$ indicates that interlayer current density $J_{l,l+1}$ between layers l and $l+1$ is proportional to $\sin(\varphi_{l,l+1})$. The periodicity of current density in a dense quasi-triangular Josephson vortex lattice imposes a constraint on $\varphi_{l,l+1}$. For layers $l = 2n$ and $l = 2n+1$, periodic conditions require:

$$\varphi_{2n-1,2n}(x+L_0)=\varphi_{2n-1,2n}(x)+2m_1\pi,$$
$$\varphi_{2n,2n+1}(x+L_0/2)=\varphi_{2n-1,2n}(x)+2m_2\pi, \tag{S12}$$

where m_1 and m_2 are arbitrary integers. Specifically we choose $m_1 = 1$ and $m_2 = 1$. Then, $P_{l,l+1}$ and $C_{l,l+1}$ satisfy:

$$P_{2n-1,2n}(x+L_0)=P_{2n-1,2n}(x),$$
$$P_{2n,2n+1}(x+L_0/2)=P_{2n-1,2n}(x), \tag{S13}$$
$$C_{2n,2n+1}=C_{2n-1,2n}+\pi.$$

We consider a particular case where $C_{2n-1,2n} = 0$ and $C_{2n,2n+1} = \pi$. Additionally, we define $C_{2n} = n\pi$ and $C_{2n+1} = (n+1)\pi$. This choice, though specific, does not lose generality since it captures the essential features of the oscillation and phase relationships in the system. To determine the form of the oscillation term P_l, we vary the free energy $F_{s,\varphi}$ in Eq. (S11) with respect to P_{2n-1} and P_{2n},

$$\frac{\partial^2 P_{2n-1}}{dx^2}+\frac{1}{\lambda_J^2}\left[\sin\left(\frac{2\pi x}{L_0}+P_{2n-2,2n-1}+\pi\right)-\sin\left(\frac{2\pi x}{L_0}+P_{2n-1,2n}\right)\right]=0,$$
$$\frac{\partial^2 P_{2n}}{dx^2}+\frac{1}{\lambda_J^2}\left[\sin\left(\frac{2\pi x}{L_0}+P_{2n-1,2n}\right)-\sin\left(\frac{2\pi x}{L_0}+P_{2n,2n+1}+\pi\right)\right]=0. \tag{S14}$$

By neglecting $P_{l-1,l}$ in sine terms, we arrive at a compact analytical expression for P_l (l includes both odd and even cases),

$$P_l=(-1)^l\frac{L_0^2}{2\pi^2\lambda_J^2}\sin\left(\frac{2\pi x}{L_0}\right). \tag{S15}$$

This ultimately leads to the order parameter phase presented in Eq. (5) of the main text. We subsequently confirm that this approximate expression for P_l performs effectively across most physically relevant parameter regimes. The dense Josephson vortex lattice exhibits a two-layer periodicity in the out-of-plane direction, implying that $P_{2n-2,2n-1} = P_{2n,2n+1}$. Combining this condition with Eq. (S14), we obtain $P_{2n-1} = -P_{2n}$ and $P_{2n-1,2n} = 2P_{2n} = -P_{2n,2n+1}$. As a result, the second equation in Eq. (S14) simplifies to

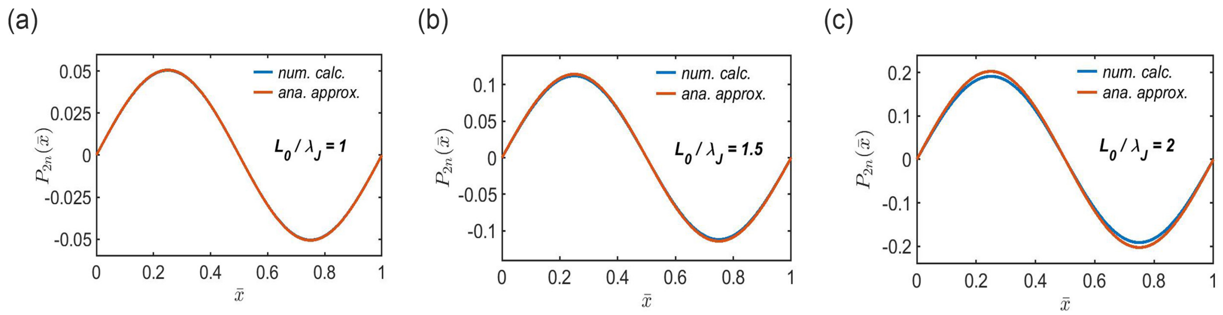

Figure S4. Comparison of numerical and approximate analytical results for the oscillation term P_{2n}. (a) $L_0/\lambda_J = 1$, (b) $L_0/\lambda_J = 1.5$, (c) $L_0/\lambda_J = 2$.

$$\frac{\partial^2 P_{2n}}{dx^2} + \frac{1}{\lambda_J^2}\left[\sin\left(\frac{2\pi x}{L_0} + 2P_{2n}\right) - \sin\left(\frac{2\pi x}{L_0} - 2P_{2n} + \pi\right)\right] = 0. \tag{S16}$$

Eq. (S16) can be further simplified and made dimensionless by introducing $\bar{x} = x/L_0$.

$$\frac{\partial^2 P_{2n}}{d\bar{x}^2} + \frac{2L_0^2}{\lambda_J^2}\sin(2\pi\bar{x})\cos(2P_{2n}) = 0. \tag{S17}$$

Eq. (S17) is the exact equation satisfied by P_{2n}. We investigate specific ratios of L_0/λ_J = 1, 1.5, 2, corresponding to B/B_P = 3.6, 2.4, 1.8. Figure. S4 shows minimal discrepancies between the accurate numerical results and the approximate analytical results, thus confirming the validity of our analytical expression.

Suppl. Note 5. Elastic moduli of the dense Josephson vortex lattice

In layered superconductors, small deformations in the x direction, $U_l^x(\mathbf{r})$, can lead to changes in the phase of the order parameter. In contrast, deformations in the y direction do not affect the phase distribution. We also neglect deformations along the z direction due to constrains imposed by superconducting layers and the invariance of the phase φ under small out-of-plane deformations. An additional term $V_l^x(\mathbf{r})$ is introduced into the phase of the order parameter, where $V_l^x(\mathbf{r}) \ll 1$ and $\mathbf{r} = (x, y)$. Notably, both $U_l^x(\mathbf{r})$ and $V_l^x(\mathbf{r})$ depend on the x and y coordinates, as the deformations in the x direction can vary non-uniformly along the y-axis due to curvature of magnetic flux lines, disrupting the uniformity of the phase in the y direction. Therefore, the phase of the order parameter φ becomes a function of $\mathbf{r}$. For simplicity, we omit the x indices in V_l and U_l. Given the weak deformation, we ensure that other terms of the order

parameter phase remain unaffected, except for the oscillation term P_l,

$$\varphi_l(\mathbf{r}) = Q_l x + S_l \pi + \tilde{P}_l(x) + V_l(\mathbf{r}), \tag{S18}$$

$\tilde{P}_l$ represents the deformed oscillation term, which satisfies the condition $\tilde{P}_l \ll 1$ for a dense Josephson vortex lattice, and we neglect the dependence of $\tilde{P}_l$ on y. P_l in Eq. (5) of the main text serves as the zeroth-order approximation of $\tilde{P}_l$. Substituting the phase expression into Eq. (2) of the main text, we can obtain the phase-dependent free energy $F_{s,\varphi}$

$$\begin{aligned} F_{s,\varphi} =& \frac{H_c^2 d}{4\pi} \left|\bar{f}\right|^2 \sum_l \int d^2\mathbf{r} \left\{ \xi_{ab,0}^2 \left[\left| \frac{d\tilde{P}_l}{dx} \right|^2 + \left| \frac{dV_l}{d\mathbf{r}} \right|^2 \right] \right. \\ & \left. -2\frac{\xi_{ab,0}^2}{\lambda_J^2} \cos\left[\frac{2\pi x}{L_0} + S_{l,l+1}\pi + \tilde{P}_{l+1} - \tilde{P}_l + V_{l+1} - V_l \right] \right\}, \end{aligned} \tag{S19}$$

In strong magnetic fields, the spatial modulation of the superconducting order parameter's modulus f becomes negligible. We replace the modulus with an average value $\bar{f}$ derived from Eq. (6). The integral of the product term $\frac{d\tilde{P}_l}{dx}$ and $\frac{dV_l}{d\mathbf{r}}$ vanishes because it contains the term $\cos(2\pi x/L_0)$, which equals zero after performing the spatial integration, while the integrals of their respective square terms remain finite. Upon expanding the cosine term, we obtain:

$$\begin{aligned} F_{s,\varphi} =& \frac{H_c^2 d}{4\pi} \left|\bar{f}\right|^2 \sum_l \int d^2\mathbf{r} \left\{ \xi_{ab,0}^2 \left[\left| \frac{d\tilde{P}_l}{dx} \right|^2 + \left| \frac{dV_l}{d\mathbf{r}} \right|^2 \right] \right. \\ & \left. +2\frac{\xi_{ab,0}^2}{\lambda_J^2} \sin\left(\frac{2\pi x}{L_0} + S_{l,l+1}\pi + V_{l+1} - V_l \right) \left(\tilde{P}_{l+1} - \tilde{P}_l\right) \right\}. \end{aligned} \tag{S20}$$

The product term containing cosine in the above equation is eliminated since $\cos\left(\tilde{P}_{l+1} - \tilde{P}_l\right) \sim 1$, and the terms $l = 2n - 1$ and $l = 2n$ cancel each other out. By varying $F_{s,\varphi}$ with respect to $\tilde{P}_l$, we obtain the expression for $\tilde{P}_l$,

$$\tilde{P}_l = \frac{(-1)^l}{(2\pi)^2} \frac{L_0^2}{\lambda_J^2} \left[\sin\left(\frac{2\pi x}{L_0} + V_{l+1} - V_l \right) + \sin\left(\frac{2\pi x}{L_0} + V_l - V_{l-1} \right) \right], \tag{S21}$$

Substituting this expression back into Eq. (S20), we derive the free energy of the deformed Josephson vortex lattice:

$$F_{s,\varphi} = \frac{H_c^2 d}{4\pi} \left|\bar{f}\right|^2 \sum_l \int d^2\mathbf{r} \left\{ \xi_{ab,0}^2 \left|\frac{dV_l}{d\mathbf{r}}\right|^2 - \left(\frac{\xi_{ab,0} L_0}{2\pi\lambda_J^2}\right)^2 \left[\cos\left(V_{l+1} + V_{l-1} - 2V_l\right) + 1\right] \right\}. \quad \text{(S22)}$$

Since V_l represents a small perturbation in the phase of the order parameter, we expand $\cos(V_{l+1} + V_{l-1} - 2V_l)$ to second order in V_l to simplify the analysis. By subtracting the mean free energy of the phase part of Eq. (6) (static Josephson vortex lattice) from the energy in Eq. (S22), we obtain the variation of the energy ΔF introduced by deformation,

$$\Delta F = \frac{H_c^2 d}{4\pi} \left|\bar{f}\right|^2 \sum_l \int d^2\mathbf{r} \left\{ \xi_{ab,0}^2 \left|\frac{dV_l}{d\mathbf{r}}\right|^2 + \frac{1}{2} \left(\frac{\xi_{ab,0} L_0}{2\pi\lambda_J^2}\right)^2 \left(V_{l+1} + V_{l-1} - 2V_l\right)^2 \right\}. \quad \text{(S23)}$$

We proceed by performing a Fourier transformation, $V_l(\mathbf{r}) = \frac{1}{\sqrt{NS}} \sum_k V_k e^{ik_{||}\cdot\mathbf{r}} e^{ik_z lD}$, where N is the total layer count and S denotes the surface area of superconducting layers. Substituting this expression into Eq. (S23), we obtain

$$\Delta F = \frac{H_c^2}{4\pi} \epsilon \left|\bar{f}\right|^2 \sum_k \left|V_k\right|^2 \left\{ \xi_{ab,0}^2 k_{||}^2 + 2 \left(\frac{\xi_{ab,0} L_0}{2\pi\lambda_J^2}\right)^2 \left[1 - \cos\left(k_z D\right)\right]^2 \right\}. \quad \text{(S24)}$$

The $\mathbf{k}$ summation is within the first Brillouin zone. The small deformation $U_l(\mathbf{r})$ relates to the phase deformation via $U_l = \frac{(V_{l+1} - V_l) L_0}{2\pi}$. In k-space, the relationship is formulated as $U_k = \frac{V_k L_0 (e^{ik_z D} - 1)}{2\pi}$. Replacing V_k with U_k in Eq. (S24) yields:

$$\begin{aligned} \Delta F &= \frac{1}{2} \sum_k \left[c_{11} k_x^2 + c_{44} k_y^2 + c_{66} \tilde{k}_z^2\right] \left|U_k\right|^2, \\ c_{11} &= c_{44} = \frac{B^2 \epsilon}{4\pi\lambda_{ab,0}^2 \tilde{k}_z^2} \left|\bar{f}\right|^2, \quad c_{66} = \frac{\Phi_0^2 \epsilon}{32\pi^3 \gamma^4 \lambda_{ab,0}^2 D^2} \left|\bar{f}\right|^2. \end{aligned} \quad \text{(S25)}$$

$\tilde{k}_z$ is the modified wave vector component in the z direction, defined as $\tilde{k}_z = 2\sin(\frac{k_z D}{2})/D$. In Eq. (S25), we have used the definition $H_c = \frac{\Phi_0}{2\sqrt{2}\pi\xi_{ab,0}\lambda_{ab,0}}$. Here, c_{11} represents the uniaxial compression modulus in the x direction, c_{44} denotes the tilt modulus in the x-y plane, and c_{66} is the shear modulus in the x-z plane. The Josephson vortex lattice exhibits anisotropy behavior in the x-z plane, being elongated in the in-plane direction, while confined in the out-of-plane

direction by superconducting layers. Compared to the hexagonal Abrikosov vortex lattice, the symmetry of the Josephson vortex lattice in the x-z plane is reduced, displaying characteristics typical of the monoclinic crystal system. According to elasticity theory, the full force constant can be expressed as $\Phi_{xx}(\mathbf{k}) = c_{11}k_x^2 + c_{44}k_y^2 + c_{66}\tilde{k}_z^2 + c_{16}k_x\tilde{k}_z$. Eqs. (S19) - (S25) demonstrate that the elastic modulus c_{16} can be neglected, allowing the calculation to be simplified to include only three elastic moduli.

Suppl. Note 6. Combined thermal and quantum fluctuations-induced melting

In the main text, we consider that the displacement of the Josephson vortice is primarily driven by thermal fluctuations, which shows agreement with experimental results. However, quantum fluctuations can also influence the Josephson vortex vibrations. In this section, we focus on calculating the melting induced by combined thermal and quantum fluctuations, and compare these results with those induced by thermal fluctuations alone.

we apply the fluctuation-dissipation theorem [6], which connects the system's response to external disturbances with its fluctuations. We define the time-domain correlation function $K(\tau)$ as

$$K(\tau) = \frac{1}{2}\langle \hat{u}(t+\tau)\hat{u}(t) + \hat{u}(t)\hat{u}(t+\tau)\rangle . \tag{S26}$$

Here, $\hat{u}$ represents displacement operator at time t, and τ is the time interval. The angle brackets denote an average over all vortices, reflecting the average behavior of the vortex lattice. The mean square displacement is calculated from the correlation function by setting $\tau = 0$:

$$\left\langle u^2 \right\rangle = K(0) = \langle \hat{u}(t)\hat{u}(t)\rangle . \tag{S27}$$

$K(\tau)$ can be expanded in Fourier form as

$$K(\tau) = \int_{-\infty}^{\infty} S(\omega)e^{-i\omega\tau}d\omega. \tag{S28}$$

Importantly, the Fourier component $S(\omega)$ can be calculated using the fluctuation-dissipation theorem

$$S(\omega) = \frac{\hbar}{2\pi} \mathbf{Im}\, G(\omega) \coth\left(\frac{\hbar\omega}{2k_B T}\right), \tag{S29}$$

where $G(\omega)$ is the response function.

We consider the Josephson vortex lattice displacement by incorporating both the pinning and damping effects between the superconducting layers [7]. Assuming these two forces are proportional to velocity $\dot{u}$ and displacement u, respectively. The total force f on vortices is:

$$f_{\mathbf{k}} = \Phi_{xx}(\mathbf{k})u_{\mathbf{k}} + \alpha_L u_{\mathbf{k}} + \eta \dot{u}_{\mathbf{k}} = [\Phi_{xx}(\mathbf{k}) + \alpha_L - i\omega\eta]\, u_{\mathbf{k}}. \tag{S30}$$

Here, $\Phi_{xx}(\mathbf{k})$ is the force constant, α_L represents the Labusch parameter mentioned in the main text, and η is the damping coefficient [8]. We define $\Phi'_{\mathbf{k}} = \Phi_{xx}(\mathbf{k}) + \alpha_L - i\omega\eta$, whose inverse, $(\Phi'_{\mathbf{k}})^{-1}$, relates to the response function $G(\omega)$ in Eq. (S29),

$$G(\omega) = \sum_k G(\omega, k) = \sum_k (\Phi_{xx}(\mathbf{k}) + \alpha_L - i\omega\eta)^{-1}. \tag{S31}$$

The summation is performed over the first Brillouin zone. The combination of Eqs. (S27) - (S31) yields the expression for the mean square displacement,

$$\langle u^2 \rangle = \frac{\hbar}{2\pi} \mathbf{Im}\left[\int_{-\infty}^{\infty} d\omega \coth\left(\frac{\hbar\omega}{2k_B T}\right) \int_{-\infty}^{\infty} \frac{dk_y}{\pi} \int_{BZ} \frac{d^2\mathbf{k}}{(2\pi)^2} \frac{1}{c_{11}k_x^2 + c_{44}k_y^2 + c_{66}\tilde{k}_z^2 + \alpha_L - i\omega\eta}\right]. \tag{S32}$$

For $\omega < 0$, we apply the transformation $\omega \to -\omega$, and Eq. (S32) is converted to:

$$\begin{aligned}\langle u^2 \rangle =& \frac{\hbar}{2\pi} \int_0^{\infty} d\omega \coth\left(\frac{\hbar\omega}{2k_B T}\right) \int_{-\infty}^{\infty} \frac{dk_y}{\pi} \int_{BZ} \frac{d^2\mathbf{k}}{(2\pi)^2} \frac{2\omega\eta}{(c_{11}k_x^2 + c_{44}k_y^2 + c_{66}\tilde{k}_z^2 + \alpha_L)^2 + \omega^2\eta^2} \\ =& D^2 \frac{\gamma}{2\pi^3 C_0} \frac{\rho_c}{R_Q} \int_0^{\infty} d\omega \coth\left(\frac{\hbar\omega}{2k_B T}\right) \int_{-\infty}^{\infty} \frac{dk_y}{\pi} \frac{1}{N_k} \sum_{i=1}^{N_k} \frac{2\omega\eta^2}{(c_{11}k_{i,x}^2 + c_{44}k_y^2 + c_{66}\tilde{k}_{i,z}^2 + \alpha_L)^2 + \omega^2\eta^2}\end{aligned} \tag{S33}$$

The viscous drag coefficient η between superconducting layers is given by $\eta = \frac{C_0 \Phi_0 B}{\rho_c \gamma D^2 c^2}$ [9], where C_0 is a constant equal to 0.3543, c is the speed of light, and ρ_c is the normal state resistivity in the out-of-plane direction. The quantum resistance R_Q is defined by the fundamental constants

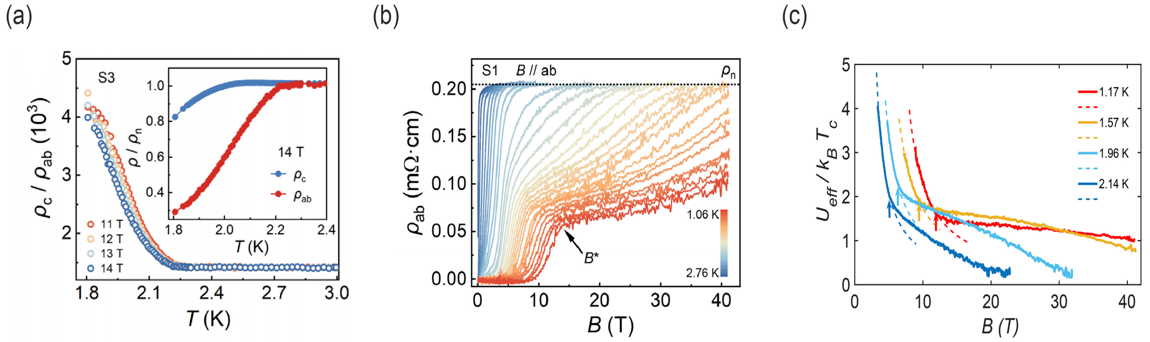

Figure S5. Anisotropic transport behavior under varying magnetic fields and temperatures. (a) Temperature dependence of the resistivity ratio ρ_c/ρ_{ab} versus temperature at different in-plane fields. The inset shows the temperature-dependent normalized resistivity ρ/ρ_n along the ab plane and c axis at 14 T. (b) Magnetic field dependence of the in-plane resistivity ρ_{ab} at different temperatures. (c) Calculated out-of-plane barrier height at various temperatures, based on the data in panel (b). The barrier height exhibits a distinct two-step behavior. Arrows indicate the melting points, while dashed lines represent the fitted inverse proportionality to the magnetic field prior to melting. The transition temperature T_c = 2.76 K. Panels (a) and (b) are reproduced from Ref. [10].

as $\hbar/e^2$. To prevent divergence in the frequency integral, we introduce a high-frequency cutoff $\omega_{\max}$ in Eq. (S33), replacing the upper limit of infinity.

In addition to the adjustable parameters α_L and $\lambda_{ab,0}(0)$, this regime also includes two additional tunable parameters, ρ_n and $\hbar\omega_{max}/k_B T_c$. To facilitate comparison between the results of Eq. (S33) and Eq. (8) of the main text, we adjust the parameter $\hbar\omega_{max}/k_B T_c$ so that the melting lines for different resistivities at high temperature ($T = T_{c0}$) match the melting line calculated under thermal fluctuations alone. The results are shown in Fig. 5(b) of the main text, with $\hbar\omega_{max}/k_B T_c = 8.6$ for $\rho_c = 10^{-5}\,\Omega\cdot m$, $\hbar\omega_{max}/k_B T_c = 6.8$ for $\rho_c = 10^{-4}\,\Omega\cdot m$, $\hbar\omega_{max}/k_B T_c = 6.95$ for $\rho_c = 10^{-3}\,\Omega\cdot m$ and $\hbar\omega_{max}/k_B T_c = 13.25$ for $\rho_c = 10^{-2}\,\Omega\cdot m$. Although the cutoff frequency ω_{max} exhibits non-monotonicity behavior with respect to resistivity, the melting line shows a monotonic trend. We observe that higher resistivity corresponds to a lower melting line, indicating enhanced quantum fluctuations. This phenomenon can be explained by inverse relationship between resistivity and viscosity: higher resistivity corresponds to reduced viscosity, leading to weaker viscous damping effects on Josephson vortex vibrations. Therefore, the melting process occurs more readily compared to the case of low resistivity.

Suppl. Note 7. Thermally activated model and effective barrier height

As mentioned in the main text, the superconducting layers act as barriers for Josephson vortices moving in the out-of-plane direction. In analogy with the thermally activated phase-slip picture of Ambegaokar-Halperin theory [11, 12], a vortex can overcome such a barrier via thermal activation, giving rise to a finite resistance. Motivated by this framework, we parametrized the normalized resistance in terms of an effective barrier height U_{eff} experienced by vortices as:

$$\frac{R}{R_n} = \left[I_0 \left(\frac{U_{\mathrm{eff}}}{2k_B T} \right) \right]^{-2}, \tag{S34}$$

where $I_0(x)$ is the modified Bessel function of the first kind and R_n is the normal-state resistance. Within this phenomenological description, the normalized resistance is inversely correlated with the effective barrier height U_{eff}. Under an out-of-plane current, Josephson vortices moving along the in-plane directions are in the flux flow regime. The effective barrier for in-plane motion is much lower than that for motion along the out-of-plane direction. Consequently, the normalized in-plane resistance is expected to be smaller than its out-of-plane counterpart. This prediction is consistent with recent experimental results on the $Ba_6Ta_{11}S_{28}$ superlattice [10]. The inset of Fig. S5(a) shows the anisotropic transport behavior as a function of temperature at a fixed magnetic field $B = 14\,\mathrm{T}$, where the out-of-plane resistivity approaches its normal-state value at a lower temperature than its in-plane resistivity.

Experimentally, the in-plane resistance exhibits a clear two-step behavior with varying magnetic field, as shown in Fig. S5(b). We attribute this behavior to the melting of the Josephson vortex lattice. To investigate the evolution of the barrier height before and after melting, we select four representative temperatures and substitute the experimental data into Eq. (S34), the results are presented in Fig. S5(c). The effective barrier height also displays a distinct two-step behavior, which persists up to 2.14 K.

Below the melting field, the effective barrier height U_{eff} can be well fitted by the form $U_{\mathrm{eff}} = A/(B - B_0)$, where A is a fitting parameter, as indicated by the dashed lines in Fig. S5(c), demonstrating an inverse relationship. Above the melting line, however, the the potential exhibits a more linear dependence and becomes much less sensitive to increasing magnetic field. We interpret this as a result due to the transition from collective, correlated dynamics of the vortices in solid phase to more independent vortex motion in the liquid phase. In the solid phase,

the elastic response of Josephson vortex lattice leads to a strong field dependence of the effective barrier. Increasing the magnetic field enhances the elastic moduli of the Josephson vortex lattice, significantly modifying vortex interactions and causing the effective barrier height U_{eff} to decrease rapidly. After melting, the loss of long-range positional order reduces such collective effects, the motion of individual vortices in liquid state is then governed by more local energy scales, resulting in a much weaker magnetic field dependence and a more gradual reduction in the effective barrier height.

A more microscopic theoretical treatment of the dynamics of interlayer Josephson vortices in both the solid and liquid phases is beyond the scope of this work and is left for future investigation.

Supplementary references

1. Zhao D, Debbeler L, Kuhne M *et al.* Evidence of finite-momentum pairing in a centrosymmetric bilayer. Nat Phys 2023; **19**: 1599.
2. Wan PH, Zheliuk O, Yuan NFQ *et al.* Orbital Fulde-Ferrell-Larkin-Ovchinnikov state in an Ising superconductor. Nature 2023; **619**: 46.
3. Cao ZZ, Liao MH, Yan HY *et al.* Spectroscopic evidence for a first-order transition to the orbital Fulde-Ferrell-Larkin-Ovchinnikov state. arXiv:2409.00373 2024; .
4. Yuan NFQ. Orbital Fulde-Ferrell-Larkin-Ovchinnikov state in an Ising superconductor. Phys Rev Res 2023; **5**: 043122.
5. Yuan NFQ. Orbital Fulde-Ferrell state versus orbital Larkin-Ovchinnikov state. arXiv:2502.18075 2025; .
6. Kubo R. The fluctuation-dissipation theorem. Rep Prog Phys 1966; **29**: 255.
7. Schmucker R and Brandt EH. On flux pinning by point pinning centres in type-II superconductors. Phys Stat Sol (B) 1977; **79**: 479.
8. Bardeen J and Stephen MJ. Theory of the motion of vortices in superconductors. Phys Rev 1965; **140**: A1197–A1207.
9. Clem JR and Coffey MW. Viscous flux motion in a Josephson-coupled layer model of

high-T_c superconductors. Phys Rev B 1990; **42**: 6209.

10. Lin JF, Wang ZQ, Yan HY *et al.* Exceedingly large in-plane critical field of finite-momentum pairing state in bulk superlattices. arXiv:2506.16039 2025; .

11. Ambegaokar V and Halperin BI. Voltage due to thermal noise in the dc Josephson effect. Phys Rev Lett 1969; **22**: 1364–1366.

12. Tinkham M. Resistive transition of high-temperature superconductors. Phys Rev Lett 1988; **61**: 1658–1661.
